# Factors controlling Mn and Zn contents in leaves of silver and downy birch in acidified soils of Central Europe and Norway

**DOI:** 10.1007/s11356-024-31837-w

**Published:** 2024-01-09

**Authors:** Gabriela Bílková, Michaela Königová, Věra Hýlová, Jitka Elznicová, Hans von Suchodoletz, Belinda Flem, Tomáš Matys Grygar

**Affiliations:** 1 Faculty of Environment, J. E. Purkyně University in Ústí Nad Labem, Pasteurova 15, 400 96 Ústí Nad Labem, Czech Republic; 2 Institute of Inorganic Chemistry of Czech Academy of Sciences, 250 68 Řež, Czech Republic; 3 Náměstí Generála Svobody 985/23, 700 30 Ostrava, Czech Republic; 4 Geoinformatics and Remote Sensing Group, Institute of Geography, Leipzig University, Johannisallee 19a, D-04103 Leipzig, Germany; 5 Geological Survey of Norway, POB 6315 Torgarden, N-7491 Trondheim, Norway

**Keywords:** Geochemical mapping, Acidification, Soils, Cation leaching, Bedrock geology

## Abstract

In Central European mountain forests, foliar element concentrations (FECs) of manganese (Mn) in silver birch (*Betula pendula* Roth) are occasionally approximately 5000 mg kg^−1^ and can represent stress for these plants. Factors controlling the Mn FECs in silver birch in Central Europe and downy birch (*Betula pubescens* Ehrh.) in Norway have not yet been fully deciphered. In this study, the Central European silver birch specimens were sampled in 2022. The samples were analysed by X-ray fluorescence spectroscopy. Norwegian data were obtained from the literature. Mn FECs are commonly negatively correlated with magnesium and, in certain areas, with potassium. Mn FECs are simultaneously elevated with zinc (Zn), likely because of soil acidification and anthropogenic emissions. Mn FECs in birch were previously thought to be related to altitude, which was assigned to (i) downslope washes of Mn or (ii) the historical load of acid emissions. The highest Mn FECs in silver birch were found in the Harz Mountains, Germany, and have been attributed to historical atmospheric contamination and the abundance of soils on felsic silicic rocks poorly buffering acid rains. The historical emission load from iron and steel production was hypothesised to be the cause of elevated Mn and Zn FECs in the Beskid Mountains, Czech Republic. Mn FECs in birch can be used to map historical soil acidification caused by industrial emissions. Zn FECs in birches can reflect soil contamination by this element.

**Supplementary Information:**

The online version contains supplementary material available at 10.1007/s11356-024-31837-w.

## Introduction

Mountain forests in Europe and North America have been significantly damaged by soil acidification, which peaked in the mid-twentieth century. Subsequently, acid rains in Europe have been mitigated by a reduction of SOx emissions in the last decades of the twentieth century (Kopáček and Veselý 2005; Šrámek et al. 2008; Hauck et al. 2012). However, forest recovery has been slower than expected (Kupková et al. 2018), partly because NOx emissions have not declined as substantially as that of SOx (Kopáček and Veselý 2005). In this context, there is an urgent question regarding the length of time needed for the recovery of anthropogenically acidified mountain soils, as additional environmental stress that further weakens European forests can be expected under ongoing global climate change. For example, summer droughts and heat waves will likely become more frequent in the coming decades, which will be particularly unfavourable for birch (Araminienė et al. 2021) and will aggravate the consequences of the soil nutrient imbalance caused by acid rains (Lomský et al. 2013; Johnson et al. 2018; Kupková et al. 2018).

Foliar element concentrations (FECs) of both nutrients and risk elements, including excess manganese (Mn) and non-nutrient trace elements, provide information on the health status of plants (Kogelmann and Sharpe 2006; White and Brown 2010; Lomský et al. 2013; Gilliam et al. 2018; Araminienė et al. 2021). The FECs of nutrients are regulated by homeostasis within relatively narrow ranges and are required to produce all essential metalloenzymes at concentrations needed for plant health (White and Brown 2010). In contrast, the variability of Mn FECs is considerably larger (Reimann et al. 2001, 2007a; Gilliam et al. 2018; Zemunik et al. 2020; Araminienė et al. 2021; Kalliola et al. 2021; Bílková et al. 2023), ranging from essential concentrations sufficient for their health (in an order of 10 mg kg^−1^; White and Brown 2010; Lambers et al. 2015) up to 10,000 mg kg^−1^ in certain hyperaccumulating plants (Lambers et al. 2015). Most common tree species growing in temperate and boreal forests, such as Norway spruce or European beech, typically show Mn FECs in the order of hundreds to a few thousand mg kg^−1^ (Reimann et al. 2001; Kogelmann and Sharpe 2006; Houle et al. 2007; Reimann et al. 2007a; Araminienė et al. 2021). Furthermore, certain common European tree species, including the silver birch (*Betula pendula* Roth) and downy birch (*B. pubescens* Ehrh.), show Mn FECs exceeding Mn concentrations in soils (Reimann et al. 2001, 2015, 2018; Wildová et al. 2021; Bílková et al. 2023) up to levels that are potentially harmful to these species (Hrdlička and Kula 2004; Bílková et al. 2023). However, the reason for this high Mn accumulation in birches is unknown.

One consequence of soil acidification is the excessive bioavailability of Mn in affected soils and, therefore, the increased Mn content in plants growing on these soils (Kazda and Zvacek 1989; Marschner 1991; Kogelmann and Sharpe 2006; Houle et al. 2007; Reimann et al. 2007b; Wills et al. 2023). Soil acidification can also result in the dieback of sensitive tree species, such as sugar maples, caused by lethal levels of Mn uptake (Kogelmann and Sharpe 2006; Houle et al. 2007; Wills et al. 2023). Soil acidification enhances Mn uptake by plants through two primary effects: (i) leaching of the nutrient elements calcium (Ca^2+^) and magnesium (Mg^2+^) from the soil, leading to their insufficiency in plants, and (ii) mobilisation of Mn species such as Mn^2+^ in the soils (Kazda and Zvacek 1989; Marschner 1991; Heal 2001; Reimann et al. 2007a; Begley-Miller et al. 2019). These processes that lead to high Mn FECs have been found for certain smaller regions and tree species; however, the interpretation of Mn FEC datasets during these preceding studies has thus far been equivocal (Hrdlička and Kula 2004; Reimann et al. 2007a; Bílková et al. 2023), whereas certain studies have found elevated Mn FECs associated with low concentrations of certain nutrients, such as Ca, Mg, and/or phosphorus (P) (Kogelmann and Sharpe 2006; Reimann et al. 2007a; Gransee and Führs 2013; Lambers et al. 2015, 2021; Gilliam et al. 2018; Zemunik et al. 2020; De Oliveira and de Andrade 2021; Roth et al. 2022). Lambers et al. (2015, 2021) interpreted excess Mn FECs as resulting from an enhanced exudation of organic acids. Generally, due to the intricate patterns of geological, topographic, climatic, and anthropogenic controlling factors, the mutual inter-element relationships in Mn uptake have rarely been examined in detail; therefore, this topic requires further research (Reimann et al. 2007a; Kalliola et al. 2021; Araminienė et al. 2021; Bílková et al. 2023). Accordingly, Alejandro et al. (2020) and others (Gilliam et al. 2018; Zemunik et al. 2020; Bílková et al. 2023) considered Mn uptake by plants to be understudied. The high tolerance of birch trees to excess Mn (Wildová et al. 2021) renders them particularly suitable for large-scale studies on factors promoting Mn uptake by plants in nutrient-poor and degraded soils.

Birch is a typical pioneer tree in Central Europe that is abundantly found on nutrient-poor soils of temperate and boreal forests (Šrámek et al. 2008; Araminienė et al. 2021) and has systematically been planted on acidified soils, for example, in the Czech Republic before the end of the twentieth century (Hrdlička and Kula 2004; Šrámek et al. 2008). A recent study in the Czech Republic (Bílková et al. 2023) identified certain factors leading to increased Mn FECs in silver birch *(Betula pendula)*, such as low Mg FECs, certain bedrock geologies (granitic rocks), and the altitude (or topography) of the sampling sites. However, the low power of these factors complicates their identification. Therefore, to fill the knowledge gap for this species, the aim of this study is to identify the major controlling factors of Mn FECs in birch trees, which show extraordinary variability, and to better understand the consequences of historical soil acidification. To accomplish this objective, we obtained an extensive collection of *B. pendula* leaves from a broad area of Central Europe, showing varied bedrock geologies, geographic settings, and anthropogenic impacts. Additionally, extensive datasets of *B. pubescens* in Norway collected for other purposes (Reimann et al. 2007a, 2007b, 2015, 2018) were revisited to verify and generalise the conclusions based on our study of *B. pendula*. Zinc (Zn) uptake was also the focus of this study, as Zn has recently been found to proceed in a manner somehow similar to that of Mn (Wen et al. 2021), and because birch is known to accumulate excess Zn from contaminated soils (Dmuchowski et al. 2013).

## Study area and methods

### Central European study areas: geology and contamination

**Fig. 1:**
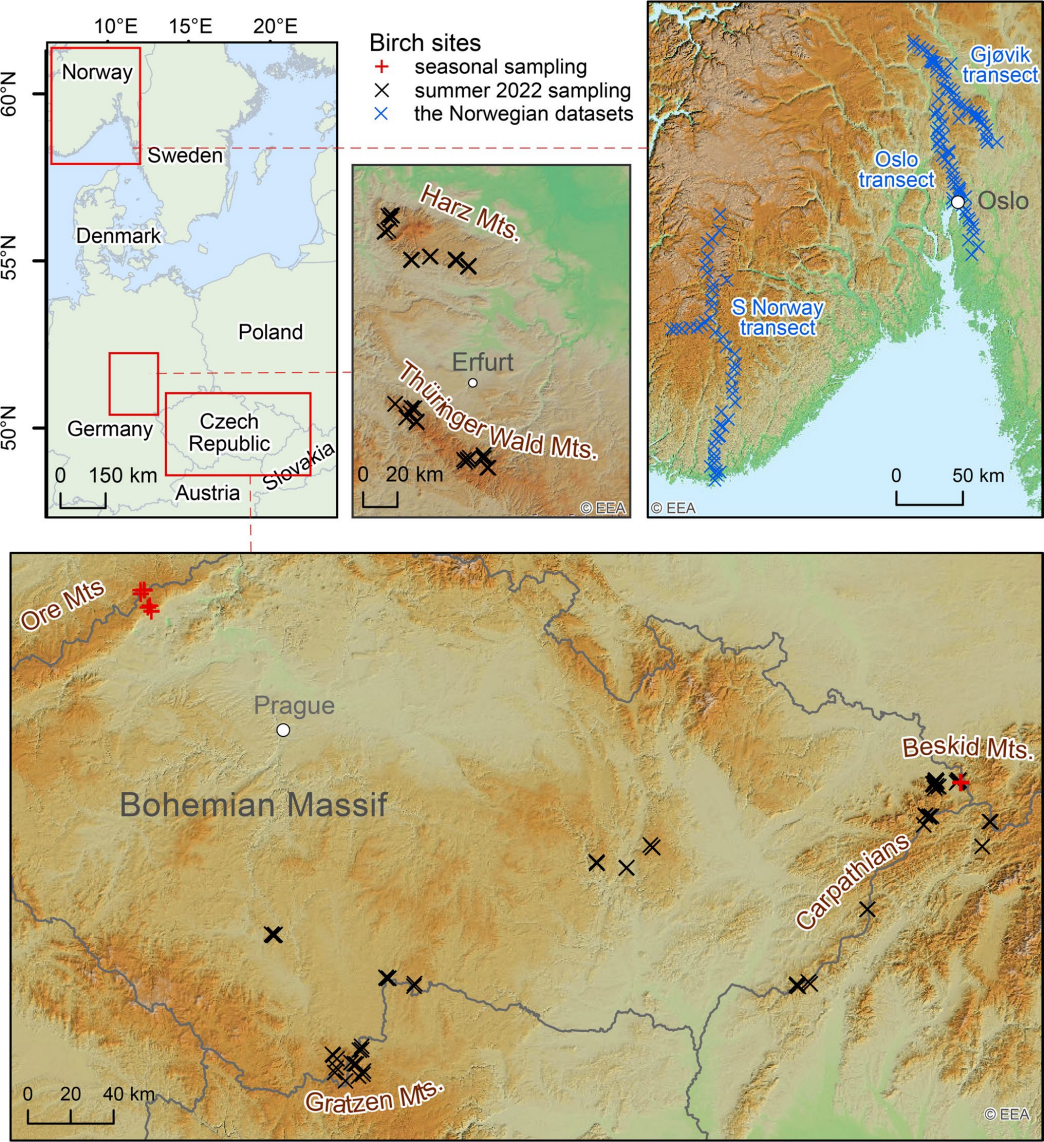
Map of the central European sampling sites

An overview of the sampling sites is provided in Tables 1 and 2 and Fig. 1. The sampling areas in Germany (Fig. 1B) and the Bohemian Massif (Fig. 1D) are located in forested hilly Hercynian Central European mid-mountain regions with mostly siliciclastic Precambrian and Palaeozoic intrusive, sedimentary, and volcanic bedrock geologies that were exhumated and uplifted during the Alpine orogeny (Embleton et al. 1984). The sites in the Carpathian Mountains, including the Beskid Mountains in the northern part (Fig. 1D), show prevalent siliciclastic Cretaceous and Cenozoic sedimentary bedrock, occasionally with minor carbonate admixtures at several sites. Areas with carbonate rocks were generally avoided because soils developed on carbonates are not preferable for birch growth.

**Table 1:** Seasonal sampling of silver birch in the Czech Republic (specimens VH and MKB)

	Number of sites	Number of trees	Interval of sampling	Number of sampling dates
Beskid Mts. (VH)	4	14	7 May–13 October	8
Ore Mts. (MKB)	7	17	23 May–28 September	6

**Table 2:** Central European areas of summer 2022 sampling of silver birch (specimens GB)

Area	Dates of sampling	Number of samples	Prevailing rocks	Emission load
Bohemian Massif	9–11 August	35	Metamorphic, volcanic, primarily felsic	Variable, diffuse
Carpathian Mts	12–13 August	18	Sedimentary, including those with glauconite, carbonate, and clays	Primarily low, diffuse
Beskid Mts. (= *northern part of Carpathian Mts*.)	14–15 August	20	Sedimentary, siliciclastic	Locally high
Harz Mts	22–23 August	26	Silicic	High (metallurgy)
Thüringer Wald Mts	24–25 August	28	Felsic volcanic; schists	Diffuse

**Fig. 2:**
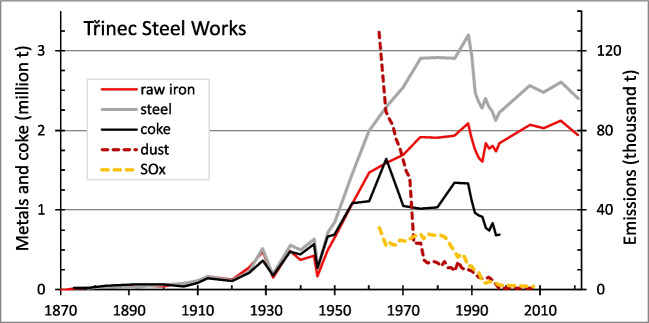
Annual production of metals, coke, and atmospheric emissions in Třinec. Compiled from Hauerová and Wawrzacz (1999), Ondraszek et al. (2009), and Moravia Steel (2022)

The sampled areas in the border region between the Czech Republic, the Slovak Republic, and Austria faced different degrees of anthropogenic SOx and NOx emissions (Krupová et al. 2018). In particular, the Beskid Mountains in the NE Czech Republic have experienced considerable recent anthropogenic impacts, as they have received significant industrial contamination and acid emissions primarily from the heavy industry in Silesia (Vácha et al. 2015; Pavlů et al. 2015; Matys Grygar et al. 2023). Generally, SOx and NOx emissions in the entire NE Czech Republic have been above average during the last decades (Krupová et al. 2018), and the primary emission source for the Beskid study area was the coke, iron, and steel production in Třinec that was established in 1839 and expanded in the second half of the twentieth century. The emission loads from Třinec peaked in the 1960s (Fig. 2), and the prevailing northerly winds transported these towards the neighbouring slopes of the Beskid Mountains, where they contributed to a massive dieback of coniferous trees in the 1970s and 1980s. Subsequently, emissions were mitigated, as shown in Fig. 2, through a rapid decline in dust and SOx.

In contrast, the Gratzen Mountains, situated along the Czech–Austrian border (Czech: *Novohradské hory*, German: *Gratzener Bergland*), have been subjected to relatively low levels of anthropogenic acid emissions during recent decades (Krupová et al. 2018); however, their soils have been naturally acidified (Pavlů et al. 2021). The Ore Mountains along the Czech–German border and the Harz Mountains in Germany have been impacted by ore mining and metal smelting (with abundant SOx emissions) since the Middle Ages, which has strongly impacted the geochemistry of local soils, waters, and sediments (Frei et al. 2000; Sharma et al. 2009; Bozau et al. 2013; Beier et al. 2022). Furthermore, during the twentieth century, the northern part of the Ore Mountains, called the ‘Black Triangle’, has been severely damaged by acid rains primarily originating from lignite power plants (Šrámek et al. 2008; Vácha et al. 2015), and the Harz Mountains have been severely impacted by acidified precipitation (Hauck et al. 2012). The Thüringer Wald Mountains have received substantially lower SOx emissions than the Ore Mountains (Zimmermann et al. 2000). Both the leachates of soils in the Harz Mountains (Hauck et al. 2012) and those from naturally acidified soils in the Gratzen Mountains have pH values ranging from three to four, falling in the range associated with aluminium buffering (Pavlů et al. 2021).

### Field work and laboratory analyses

Two sampling designs were used in 2022: one to decipher seasonal variations and the other for geographical controls. The subsets from the Beskid Mountains (VH) and Ore Mountains (MKB) were focused on the seasonal dynamics of FECs and were sampled with an approximate monthly resolution (Table 1; complete results, site characteristics, and sampling dates are shown in Tables S1 and S2). The study in the Ore Mountains also included parallel sampling at two different sides of trees, two or three different heights of a single tree, and sampling of several neighbouring trees to characterise possible intra- and inter-specimen variability under otherwise identical site conditions.

The samples labelled GB were collected from geographically and geologically diverse sites of Central Europe in August 2022 (Table 2; complete results and site characteristics are listed in Table S3) and also included the July samples from the VH and MKB subsets to identify Mn-controlling factors other than time.

Similar to previous studies (Wildová et al. 2021; Bílková et al. 2023), the leaves were sampled without washing in paper bags. The concentrations of the target elements in the leaves were high because of their accumulation relative to the soil (Reimann et al. 2007a, 2007b), and traces of soil or dust could not contaminate them, as discussed by Bílková et al. (2023). For example, Pająk et al. (2017) compared Zn FECs in washed and unwashed birch leaves from Poland and found only minor differences in mean concentrations and concentration ranges.

Laboratory elemental analysis was performed using energy-dispersive X-ray fluorescence spectrometry (XRF), as described by Bílková et al. (2023). The leaf samples were pulverised in a planetary micromill (Fritsch, Germany) and subsequently poured into Nylon cells with Mylar foil bottoms. The XRF analysis was performed using an Epsilon 3^X^ spectrometer (Malvern-Panalytical, Netherlands) equipped with an X-ray tube (Ag cathode, up to 50 kV) and a Peltier-cooled large-area Si-drift detector.

### Norwegian datasets

Reimann et al. (2007a, 2007b, 2015, 2018) published the FECs results in downy birch (*B. pubescens*) along with descriptions of the topography, climate, land cover, and geology of the study areas. The quoted studies (Table 3) addressed plant–soil interactions, corroborating the ideas proposed by Goldschmidt (1937), which were revisited by Reimann et al. (2001), comprising translocations of elements in soil profiles by complex actions of plants. However, the inter-element relationships and the impact of external factors controlling Mn and Zn FECs were not addressed in these studies.

**Table 3:** Revisited datasets for downy birch in three transects in Norway. MG represents the mean gradient of the terrain

Transect	Altitude (m)	MG (m km^−1^)	Longitude (°N)	Major geology	Reference
S Norway	10–1000	9	59.0	Gneiss	Reimann et al. (2015)
Oslo	100–550	15	60.5	Gneiss, syenite, shales	Reimann et al. (2007a, 2007b)
Gjøvik	200–800	5	60.4	Syenite, gneiss, shists, sediments (clastic, carbonatic)	Reimann et al. (2018)

The former sampling campaigns covered three transects that were named the South Norway, Oslo, and Gjøvik transects (Fig. 1B). Their characteristics are summarised in Table 3. The South Norway transect begins at the Skagerak Strait and stretches approximately 200 km inland through six vegetation zones, with a stepwise declining presence of birch towards higher altitudes farther from the sea. The climate of this transect is characterised by a mean annual precipitation (MAP) declining from approximately 1500 mm/a near the coast to approximately 1,100 mm/a inland, with mean annual temperatures (MATs) ranging from 8 to 6 °C near the coast to − 4 to − 6 °C inland. The Oslo transect is 120 km in length, begins approximately 30 km south of Oslo, crosses the city, and stretches further north. It encompasses an MAP ranging from 700 to 1000 mm and an MAT ranging from 4 to 6 °C. The Gjøvik transect is 100 km in length, its centre is proximal to the northern end of the Oslo transect, and it stretches at a slight angle diagonally to that transect (Fig. 1B). Therefore, its climate is similar to that of the Oslo transect (900 mm MAP and 4 °C MAT in Gjøvik). The Gjøvik transect shows the largest geologic variation.

### Data visualisation and processing

Tools developed for exploratory data analysis (Tukey 1977) were employed for data visualisation and processing because most environmental geochemistry datasets can be polymodal with non-Gaussian distributions and a common occurrence of outliers (Reimann et al. 2005). Empirical cumulative distribution functions (ECDFs) were used to visualise the univariate data structure as a versatile tool for identifying the boundaries between partly overlapping modes, as demonstrated by Stanley and Sinclair (1987) and Sinclair (1991). Boxplots suitable for the visualisation of data with non-Gaussian distributions (Tukey 1977) and results showing relatively weak correlations (Bílková et al. 2023) were used to examine the relationship between the Mn FECs and FECs of nutrients. To achieve this objective, the data series were separated into quartiles for nutrient concentrations, and similar to Bílková et al. (2023), Mn is shown as a Tukey boxplot for these quartiles.

Variability in the datasets was evaluated using the non-Gaussian parameters proposed by Tukey (1977) and promoted by Reimann et al. (2005), with median instead of mean, median absolute deviation (MAD) instead of standard deviation, and MAD/median instead of relative standard deviation.

Multilinear regression analysis was performed after removing outliers using Tukey’s upper fences; that is, individual extremes in the individual concentration data series were neglected because such outliers, which are common in geochemistry datasets, can considerably limit the performance of multivariate statistical analyses (Reid and Spencer 2009).

## Results

### Seasonal FEC dynamics

Seasonal FEC dynamics were studied in the Ore Mountains (MKB) and Beskid Mountains (VH) to evaluate the possible effects of unequally long growing seasons at the individual sampling sites. The complete results are shown in Tables S1 and S2, and a qualitative evaluation of the seasonal dynamics is summarised in Table 4.

**Table 4:** Seasonal changes in FECs

Element	Ore Mountains (MKB)		Beskid Mountains (VH)	
	Entire growing season	Summer	Entire growing season	Summer
Mg	Irregular	Stable	Spring minimum	Stable
P	Sprout maximum	Stable	Sprout maximum	Stable
K	Irregular	Stable	Sprout maximum	Stable
Ca	Decrease after sprout	Growth	Spring growth	Growth
Mn	Summer maximum	Growing or stable	Spring minimum	Variable
Zn	Summer maximum	Stable	Autumn maximum	Stable

However, the Mn concentration changes in the MKB and VH subsets during the summer months (July–September) were considerably less significant than those reported by Wildová et al. (2021). In the VH and MKB subsets, the median MAD/median values (equivalent to the average relative deviation) for individual trees or tree branches were only 4.7% and 5.0%, respectively, and the maximum values for individual trees were evenly distributed over the summer period. Thus, the summer variability for individual trees was considerably lower than the median MAD/median for all the VH and MKB datasets (27% and 26%, respectively). The non-significant impact of the selection of individual trees or branches was also confirmed using analysis of variance(ANOVA). The low summer variability also proves that the timing of sampling is not critical and does not influence inter-site comparisons.

### Geographic and geologic variability of Mn FECs

**Fig. 3:**
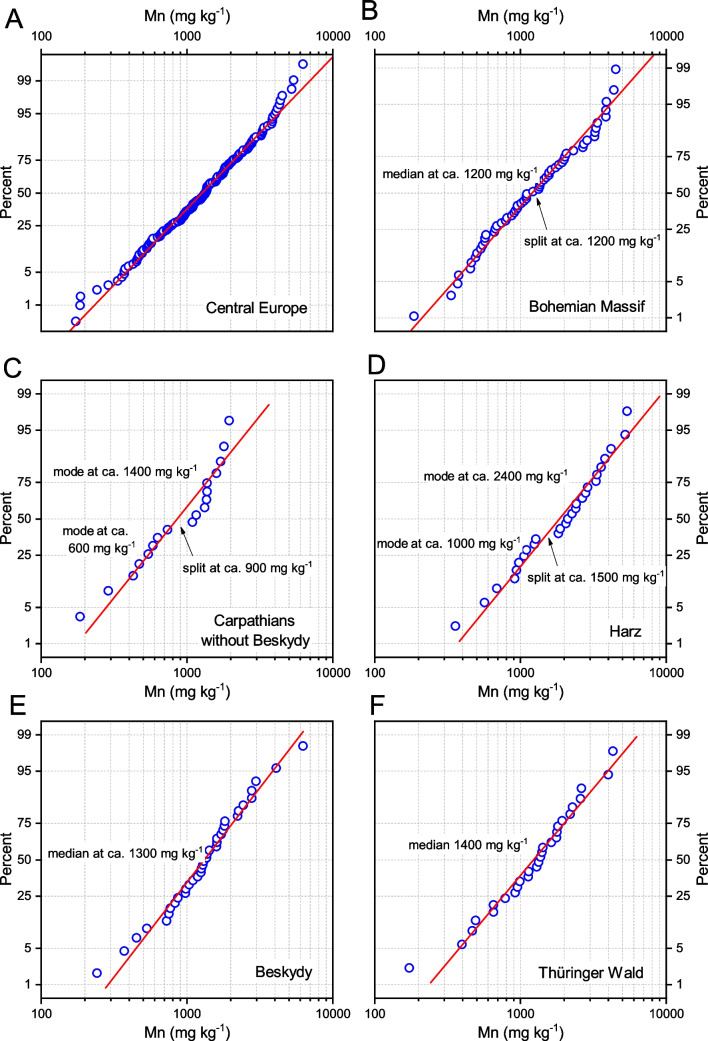
ECDFs of Mn FECs in *B. pendula* for the central European dataset (**A**), and selected geographic subsets (**B**–**F**). The red lines are fits to a log-normal distribution. Important breaks (splits in ECDFs), showing bimodal distributions, are shown by arrows

The FECs in the Central European collection (subsets GB and August samples of the VH and MKB subsets) are summarised in Table S3. The ECDFs of Mn FECs are shown in Fig. 3. The range of Mn concentrations varied considerably, with Q1 and Q3 at 800 and 2,000 mg kg^−1^, respectively, for the entire Central European dataset (Fig. 3A). The Mn concentration showed bimodal distributions in the Carpathian and Harz Mountains (Fig. 3C and D). In the Beskid and Harz Mountains, which are more influenced by acid rains and acid bedrock, Mn FEC concentrations were higher than those in the rest of the samples from the Carpathian Mountains. The low Mn FEC in the Carpathian Mountains is consistent with a prevalence of more basic bedrock and less acidic contamination. Notably, there is a gap (split) in most ECDF plots (Fig. 3) around the Mn FEC of 1000 mg kg^−1^, thus showing bimodality, particularly in the Bohemian Massif (1200 mg kg^−1^), Carpathian (900 mg kg^−1^), and Harz Mountain subsets (1500 mg kg^−1^, Fig. 3).

**Fig. 4:**
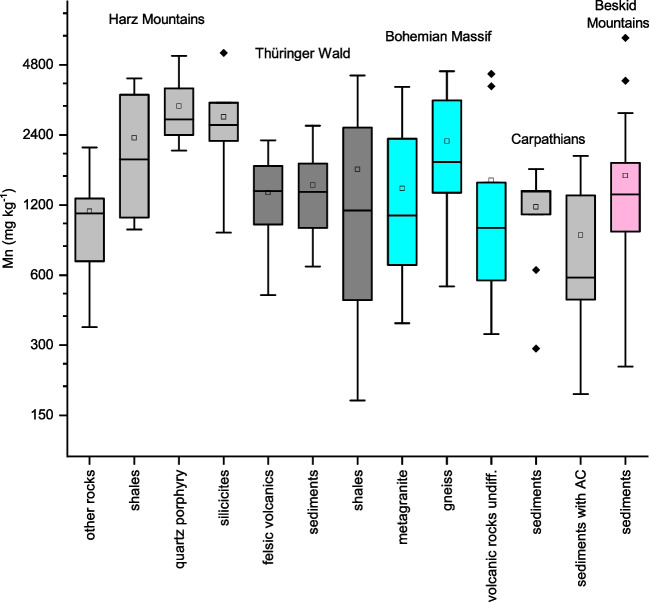
Manganese FECs in the central European dataset, separated according to geography and geology of soil forming bedrock. AC, autochthonous sedimentary components (carbonates, glauconite)

The Mn FECs on sedimentary rocks were the most variable within several datasets; the broadest interquartile ranges (that is, Q3–Q1, visualized as the box dimensions in the boxplots of Fig. 4) were found on shales in the Harz and Thüringer Wald Mountains (Fig. 4). Therefore, in these areas, additional sources of Mn FEC variability must be identified (see below).

**Fig. 5:**
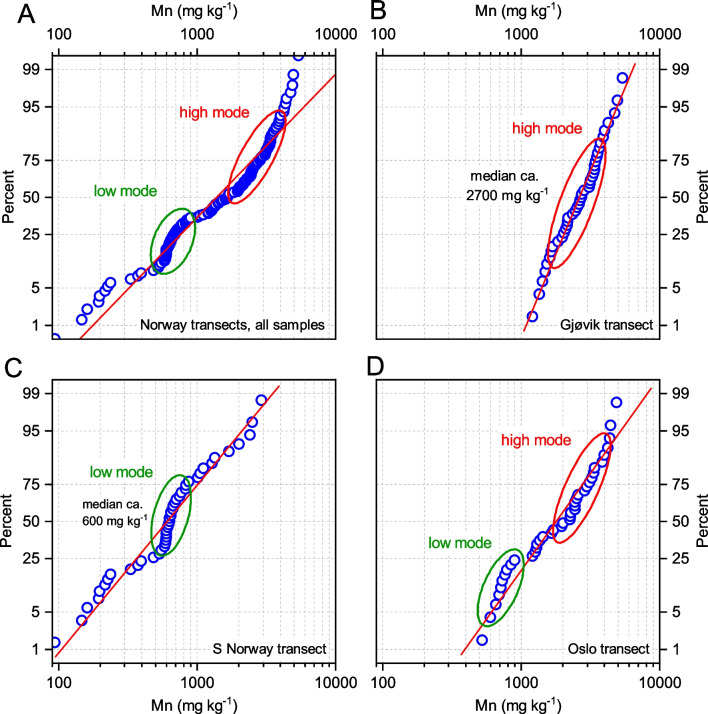
ECDF of Mn FECs in *B. pubescens* for all Norwegian datasets (**A**), and for the three individual transects (**B**–**D**). Low and high Mn concentration modes are highlighted

In the Norwegian transects, Mn FECs exhibited polymodal distributions with two primary Mn concentration modes (Fig. 5). The lower one, centred at approximately 600 mg kg^−1^, dominates the S Norway transect and comprising its entire second and third quartiles (Fig. 5C) but is less abundant in the Oslo transect (its first quartile; see Fig. 5D). In the Gjøvik transect, no Mn FECs lower than 1000 mg kg^−1^ were found, showing only the higher Mn FEC mode with a median as high as 2700 mg kg^−1^ (Fig. 5B). The primary concentration mode in the Oslo transect was centred at approximately 2400 mg kg^−1^. This polymodality cannot be attributed to geological variability, as the Gjøvik transect with the most homogeneous ECDF shows the most varied geology, while the opposite is found in the S Norway transect. These transects exhibit similar geographic conditions (Table 3).

### Inter-element relationships in FECs

Inter-element relationships were examined by multilinear regression, and the results are summarised in Table 5. In these regressions, the constant term was fixed to zero because (i) it was statistically insignificant when it was included in the regression, and (ii) it was consistent with the intercept that produced poor regressions with an *R*^2^ < 0.2. Regression analysis primarily showed positive correlations of Mn FECs with P, Ca, and Zn and negative correlations with K and Mg. In certain geographical subsets, these correlations were weak for Mg, K, Ca, and Zn (Table 5).

**Table 5:** Multilinear regression for Mn FECs (dependent variable) and the FECs of Mg, P, K, Ca, and Zn (independent variables). Regression coefficients (their errors in parentheses) are listed for the individual FECs. Abbreviations: *Carp*. Carpathian Mts., *TW* Thüringer Wald Mts., *n.s*. non-significant (Student’s *t*-test), *DF* degrees of freedom (number of samples minus the number of fitted parameters), *OL* univariate outliers excluded from the regression analysis (Tukey’s upper fence)

	2022 Central Europe dataset				Revisited Norway datasets			
	All samples	Bohemian Massif	Carpathians + Beskids	Harz + TW	All samples	S Norway	Oslo	Gjøvik
Mg	− 0.34 (0.13)	− 0.63 (0.21)	n.s	− 0.31 (0.20)	− 0.85 (0.11)	− 0.36 (0.12)	− 0.63 (0.29)	− 0.41 (0.31)
P	0.43 (0.08)	0.59 (0.13)	0.28 (0.09)	0.42 (0.11)	0.57 (0.13)	0.53 (0.10)	0.48 (0.24)	0.49 (0.28)
K	− 0.14 (0.04)	− 0.26 (0.07)	− 0.11 (0.05)	n.s	n.s	− 0.09 (0.03)	n.s	n.s
Ca	0.09 (0.04)	0.34 (0.08)	n.s	n.s	0.34 (0.05)	0.17 (0.06)	0.19 (0.12)	0.20 (0.11)
Zn	2.84 (0.77)	n.s	3.58 (0.89)	3.00 (1.49)	3.61 (0.99)	2.74 (1.24)	4.87 (1.63)	5.31 (2.53)
*DF*	159	53	50	51	118	40	33	35
OL	3	1	1	1	4	0	3	3
*R*^2^	0.70	0.73	0.73	0.72	0.83	0.80	0.79	0.89

**Fig. 6:**
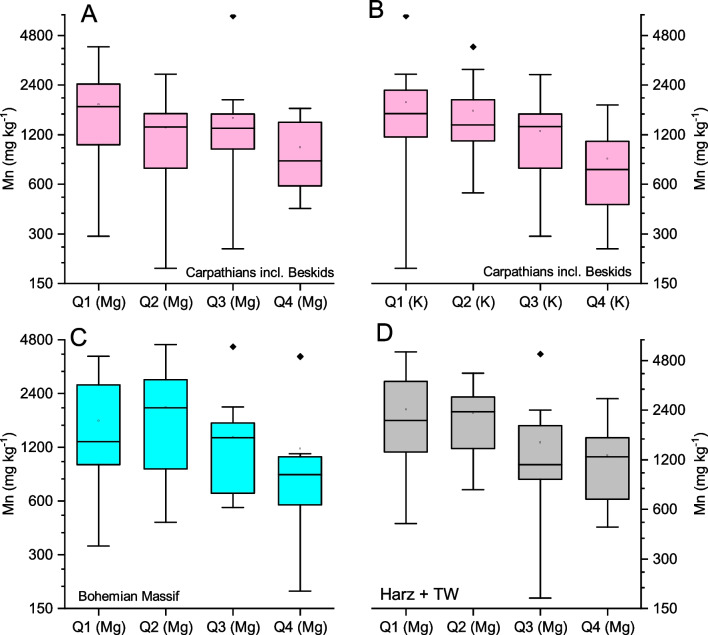
Boxplots of Mn FECs separated to quartiles according to Mg and K FECs in selected subsets of the central European dataset

The negative correlations of (i) Mn and Mg and (ii) Mn and K were visualised using boxplots for the Central European datasets (Fig. 6). The trend of decreasing Mn FECs with increasing Mg for the Carpathians, including the Beskids, is shown in Fig. 6A, although this was not significant in the corresponding multilinear regression analysis (Table 5). Similarly, Fig. 6B shows a decreasing trend of Mn FECs with increasing K in this mountain range. Furthermore, Fig. 6C and D show non-monotonous trends of the Mn concentration medians with increasing Mg for the Bohemian Massif and Harz/Thüringer Wald Mountains; therefore, generally lower Mn FECs can be expected for higher Mg FECs, corresponding to the negative correlation between Mg and Mn (Table 5).

**Fig. 7:**
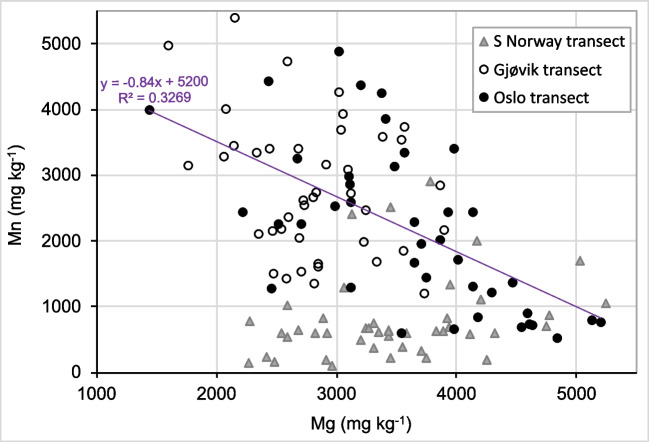
Scatterplot of Mn versus Mg FECs in downy birch in the Norwegian transects. The joint regression line and equation (violet coloured) for the Oslo and Gjøvik transects (coalesced) are shown as well

In Norway, also for the Oslo and Gjøvik transects, the birch FECs showed weak negative relationships between Mn and Mg (*R*^2^ of 0.33 for both unified transects) (Table 5, Fig. 7). In contrast, no such relationship was found in the S Norway transect, although similar bedrock geology and climatic conditions, as well as the same range of Mg FECs, were found. In this context, notably, the Mn FECs in the latter transect primarily belonged to the lower primary mode of < 1000 mg kg^−1^ (Fig. 5C). This equivocality of the possible Mg control on Mn FECs demonstrates that the inter-element relationships in FECs are not universal but seem to be site-specific. This could explain why the factors controlling Mn FECs have not been identified in previous studies.

### Impact of elevation and topography on Mn FECs

**Fig. 8:**
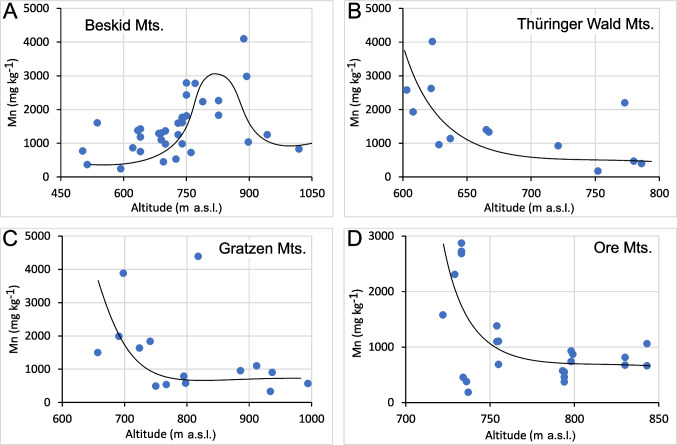
Dependence of Mn FECs on the altitude of the sampling sites for the Beskid Mountains (**A**, one high outlier not shown), sites on shales, phyllite, and greywacke in the Thüringer Wald (**B**), the Gratzen Mountains (**C**), and the Ore Mountains (**D**). The curves were drawn ‘by hand’ to highlight the elevation trends

The Mn FECs in several subsets with broad ECDFs were weakly controlled by altitude and/or topography of the sampling sites (Fig. 8). Table 6 shows the results of the ordinary regression analysis of the data shown in Figs. 8B–D, confirming the influence of elevation. In the Beskid (Fig. 8A) and Gratzen Mountains (Fig. 8C), the bedrock geology is uniform (siliciclastic sediments and granites + gneiss, respectively). From the Thüringer Wald collection (Fig. 8B), we exclusively selected sites located in the eastern part of the study area with siliciclastic sediments (greywacke, phyllite, and shale) to examine only the altitudinal control without introducing bias from varying bedrock geology. Altitudinal control in the Beskid, Thüringer Wald, and Ore Mountains was accompanied by inverse proportionality of Mg and Mn FECs; that is, the Mg FECs showed altitudinal control in these areas also. In the Beskid Mountains (Fig. 8A), the Mn FEC maxima were found in the elevation range of 750–900 m a.s.l. In all the other areas (Fig. 8B–D), higher Mn FECs were more frequent at lower elevations, whereas minimal values were primarily observed at higher altitudes.

**Table 6:** Results of regression analysis of the data shown in Fig. 8, including Student’s *t*-test probability (*P*) values

Data	Linear regression equation; regression coefficient
Thüringer Wald Mts. (Fig. 8B)	Mn = − 9.27·elevation + 7873; *R*^2^ = 0.36 (*P* > 99%)
Gratzen Mts. (Fig. 8C)	Mn = − 4.86·elevation + 5137; *R*^2^ = 0.31 (one point removed, *P* = 98%)
Ore Mts. (Fig. 8D)	Mn = − 9.58·elevation + 8508; *R*^2^ = 0.23 (*P* = 95%)

**Fig. 9:**
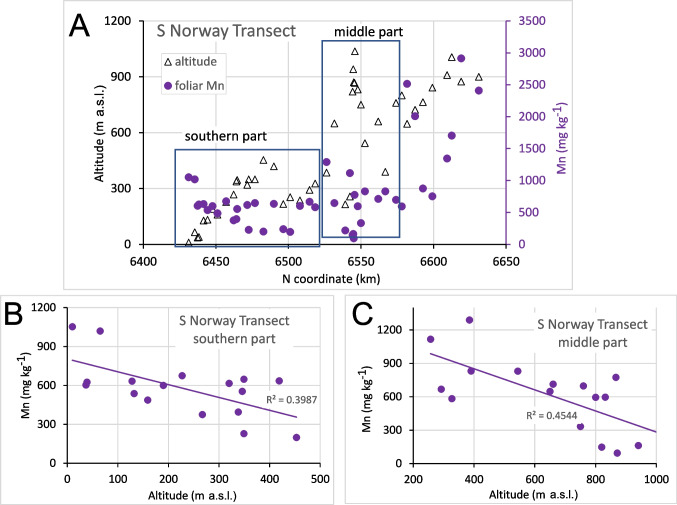
Altitude of the sampling sites and Mn FECs in *B. pubescens* in the S Norway transect (**A**), and relationships between Mn FECs and altitude in the southern (**B**) and middle part (**C**) of this transect

Certain relationships between Mn FECs and altitude or topography were also found in the S Norway and Gjøvik transects, where neither bedrock geology nor inter-element relationships explained the observed differences in Mn FECs (Fig. 5). The Mn FECs were highest and least scattered in the Gjøvik transect (Fig. 5B), which showed the lowest effective altitudinal range of the three examined Norwegian transects. Although the overall altitudinal range in this transect was high (200–800 m a.s.l.; Table 3), half of the samples were obtained between 380 and 540 m a.s.l., and the mean slope between neighbouring sites was the lowest of the three transects (5 m km^−1^; Table 3). The lowest Mn FECs were found in the S Norway transect, which showed a nearly continuous slope in its three segments (Fig. 9A), with a general slope trend from the sea coast to nearly 1000 m a.s.l. (Table 3). Larger Mn FECs were found at lower slopes and lower concentrations at elevated sites. This increase at lower elevation (Fig. 9) can be explained by the downslope transport of soil Mn^2+^.

### Zn FECs

**Fig. 10:**
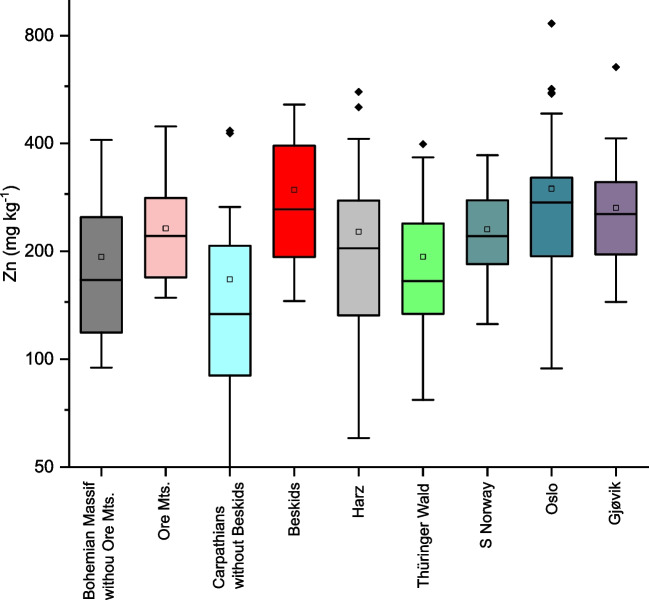
Boxplots of Zn FECs in the central European subsets and Norway transects

The joint uptakes of Mn and Zn were documented by correlation of their FECs (Table 5). Figure 10 shows the highest median and third quartile of the Zn FECs for *B. pendula* in the subset of the Beskid Mountains with the areas surrounding the Třinec Steel Works, which have been contaminated from metallurgical activities with several risk elements, including Zn (Fig. 2). Relatively high levels of foliar Zn were found in Norwegian samples of *B. pubescens*. Foliar Zn was particularly high in the S Norway transect, where contamination surrounding Oslo was reported by Reimann et al. (2007b). Zn contents were also elevated in the Gjøvik transect, where Mo and Pb mineralisation impacted element contents in plants (Reimann et al. 2018).

## Discussion

### Evaluation of factors controlling Mn FECs in birch trees

The results of the inter-element correlations are summarised in Table 5 and Figs. 6 and 7. On the one hand, the negative correlation of Mn and Mg aligns with most previous findings summarised in the ‘Introduction’, and the positive correlation of Mn and P agrees with the model of joint uptake of both elements by low-molecular complexing acids (Lambers et al. 2015; 2021). On the other hand, Araminienė et al. (2021) found a negative correlation between P and Mn FECs, underlining the uncertainties in inter-element relationships in FECs. We found positive correlations between Mn, Ca, and Zn (Table 5) in our datasets, while a negative correlation between Mn and Ca was found by Araminienė et al. (2021). These discrepancies document the complexity of Mn uptake by birch trees and indicate that more controlling factors exist than those considered in this study.

Wildová et al. (2021) reported considerable seasonal dynamics of Mn FECs in birch. Such temporal variability of Mn FECs would complicate the comparison of Mn FECs within geographically broader areas and sites of variable altitudes and limit the applicability of geochemical mapping with birch leaves. However, this study (Table 4, S1, and S2) shows that birch Mn FECs in summer were sufficiently stable; thus, the sampling date during summer should not be critical for inter-site comparison. The two most common FEC trends were (i) a peak during sprouting followed by relatively stable concentrations in summer and occasionally a decrease before senescence, which is typical for K and P, and (ii) a seasonal (or at least summer) increase in Ca, Mn, and Zn. Both patterns are similar to those reported in previous studies (Tamm 1951; Duchesne et al. 2001; Piczak et al. 2003; Wildová et al. 2021; Kalliola et al. 2021).

The impact of the bedrock geology was examined as a possible factor causing bimodal or polymodal Mn distributions in different geographic areas. Figure 4 shows the Mn FECs in the Central European subsets, which were separated according to their major types of soil-forming bedrock. However, provided that all study areas showed specific suites of soil-forming bedrock (Table 2), it was not possible to completely separate the geographic and geological controlling factors. Geologic control is most distinct in the Harz Mountains subset, where low Mn FECs were found on alkali rhyolite and greywacke (aggregated as ‘other rocks’ in Fig. 4), while high Mn FECs were found on siliceous bedrocks (locally abundant quartz porphyry and quartzites). Such a pattern could be expected because soils on siliceous bedrock are more prone to acidification and, therefore, poorer in nutrient cations than soils on alkaline volcanic rocks or geochemically immature greywackes. Similarly, in the Bohemian Massif dataset, higher Mn FECs were found on gneiss than on volcanic rocks (Fig. 4), although the geological control was less significant than that in the Harz Mountains. The lowest median of Mn FECs in the Central European dataset was found in the subset of soils on sedimentary rocks with autochthonous components (carbonate and glauconite) in the Carpathian Mountains (Fig. 4) because these autochthonous components provide abundant nutrient cations for plants and sufficient bases to prevent soil acidification.

The impact of sampling site elevation (Figs. 8 and 9) can be partly explained by the downslope transport of Mn^2+^, as low Mn FECs were generally found at higher elevations of the S Norway transect with steeper terrain. In contrast, due to its generally flat terrain, no downslope transport (wash out) of Mn should be expected in the Gjøvik transect, which would explain generally higher Mn FECs in this transect. The slope effect in the S Norway transect resembled the observations in three of the four cases shown in the Central European GB dataset (Fig. 8B–D). Similarly, downslope transport was previously also assumed by Bílková et al. (2023) to explain the highest thus far reported Mn FECs of the Ore Mountains in a downslope position at approximately 550 m a.s.l. on the Czech side near Litvínov (Hrdlička and Kula 2004; Wildová 2022).

### Association of Zn and Mn FECs

Under similar environmental conditions, next to Mn, Zn is another micronutrient whose uptake by birch is larger than that by most other common forest plants, apart from willows (Reimann et al. 2001, 2007b, 2015). Joint (correlated) uptake of Mn and Zn by birch, as shown in our data (Table 5), has not been reported in previous field studies. Both Mn and Zn are more mobile in acid soils (Frei et al. 2000; Bozau et al. 2013; Pavlů et al. 2015). Therefore, the association of Mn and Zn FECs in birch could primarily be attributed to soil acidification and/or the joint mobilisation of both nutrients by carboxylate exudates (Wen et al. 2021). Additionally, both Zn and acid emissions originate from metallurgical activities, and the historical smelting of silver and lead from polymetallic ores using the oxidising roasting method produces significant emissions of SOx and volatile risk elements, including Zn. With respect to the Harz Mountains, a crucial historical silver smelter is located in Goslar on the northern slope of the mountains (Sharma et al. 2009), and several other smelters of non-ferrous metals are situated along their northern slopes (Fig. S1). Furthermore, atmospheric contamination with SOx and its associated risk elements is associated with iron and steel production. Accordingly, the agricultural soils surrounding the Třinec Steel Works are contaminated by Zn (Pavlů et al. 2015; Vácha et al. 2015; Matys Grygar et al. 2023).

### Consequences of soil Mn mobilised by acidification

**Fig. 11:**
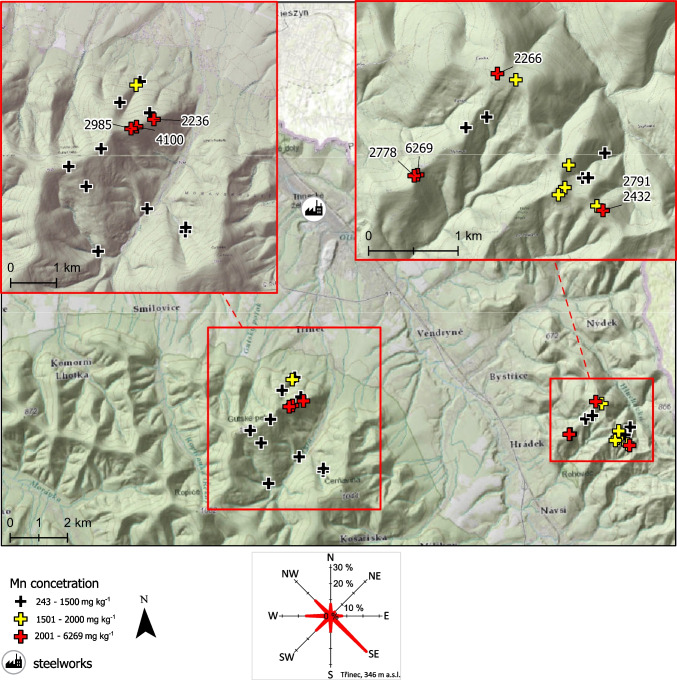
Manganese FECs in silver birch in the surroundings of Třinec, with the highest values found on the hills and ridges exposed to the Iron and Steel Works. Windrose shows average frequency of wind directions in period of 2015–2022 (Czech Hydrometeorological Institute)

The causality of soil–plant interactions cannot easily be deciphered because they are characterised by excessive feedback (Gilliam et al. 2018; Begley-Miller et al. 2019). Primarily, increased solubility (bioavailability) of soil Mn due to acidification enhances damage to plant communities (Reimann et al. 2007a, 2007b; Zemunik et al. 2020) and can even cause dieback of sensitive species (Kogelmann and Sharpe 2006; Houle et al. 2007). Consequently, soil acidification leads to a preference for plants tolerant of excess Mn (Hauck et al. 2002; Begley-Miller et al. 2019; Roth et al. 2022). Accordingly, strong soil acidification prompted the decision made by Czech forest managers to replace dying Norwegian spruce plantations in the Ore Mountains with more Mn-tolerant birches before the end of the twentieth century (Šrámek et al. 2008). This tolerance, based on Mn accumulation in leaves, causes biotic cycling of this element such that it remains in its bioavailable form (Mn^2+^) (Navrátil et al. 2007; Reimann et al. 2007a; Gilliam et al. 2018; Wildová et al. 2021). In litter (senesced leaves), Mn is present as a soluble organic complex that cannot be oxidised to insoluble Mn^III,IV^ oxides under generally reducing conditions in organic matter-rich topsoil horizons; thus, Mn is kept in the biotic cycle. Wills et al. (2023) noted the phytotoxicity of Mn on tree species in the north-eastern U.S. as a ‘persistent legacy of soil acidification’. Therefore, Mn bioavailability is likely a consequence of historical soil acidification, and the subsequent success of Mn-accumulating plants will likely slow the recovery of acidified forest soils. Slow forest recovery has been documented in the Ore Mountains (Kupková et al. 2018), elsewhere in Europe (Johnson et al. 2018), and in the USA (Wills et al. 2023). Accordingly, the Beskid Mountains subset (Figs. 8A and 11) demonstrated the persistence of the historical emission signal from the mid-twentieth century, although, after the 1970s, contamination was considerably limited due to technological improvements (Fig. 2).

High Mn mobility in acidified soils can result in downslope transport of Mn^2+^ (Heal 2001). Accordingly, we found elevated Mn FECs in birch growing at lower downslope altitudes in the Harz (Fig. 8B), Gratzen (Fig. 8C), and Ore Mountains (Fig. 8D), as well as along the slopes of the S Norway transect (Fig. 9). Similarly, Hrdlička and Kula (2004) and Wildová (2022) also found downslope increasing Mn FECs of birch in the Ore Mountains.

### Can birch leaves be used for geochemical mapping?

The Mn FECs in birch leaves can serve as a proxy for both soil acidification and specific contamination associated with heavy industries. This was demonstrated in a study by Ernst and Nelissen (2008), who found a positive correlation between Mn FECs and risk elements (Cu and Zn) in birch leaves and between Mn FECs and chemically reactive concentrations of both risk elements in soils. Accordingly, Ernst and Nelissen (2008) and Dmuchowski et al. (2013) proposed the use of birch leaves for environmental monitoring. Furthermore, a correlation between foliar and soil Mn was found for maple (Kogelmann and Sharpe 2006) and birch in a Norwegian transect studied by Reimann et al. (2015), and enhanced Mn FECs were linked to low nutrient concentrations (Mg and K; Table 5), which are associated with soil acidification (Šrámek et al. 2008). Therefore, the Mn FECs in birch are promising proxies for environmental geochemical mapping, although this has not yet been realised.

Accordingly, the impact of the Třinec Steel Works on Mn and Zn FECs in the Beskid Mountains, as discussed above, is a promising example for the use of birch for geochemical mapping, as higher Mn FECs were found in local slopes and ridges exposed to atmospheric input from Třinec (Figs. 8A and 11). Notably, no simple downslope increase of foliar Mn was observed in the Beskids. This can be attributed to the relatively short and recent impact of acidification (Fig. 2), which has not yet resulted in the overall downslope wash of Mn observed in the persistently acid soils of the other study areas.

Excess Mn FECs in birch can also indicate soil conditions that potentially endanger sustainable tree growth. The Mn FEC maxima in the studied datasets of approximately 5000 mg kg^−1^, also found by Bílková et al. (2023), were significantly higher than the actual plant requirements. While birch can adapt to such high FEC concentrations by Mn^2+^ sequestration at the subcellular level (Alejandro et al. 2020; De Oliveira and de Andrade 2021) and storage of excess Mn at the leaf edges (Kalliola et al. 2021), this mechanism is not effective for other plant species. While these plants can cope with soil contamination by several other mechanisms, such as the depth of rooting (Reimann et al. 2007a) or uptake of nutrients detoxifying Mn (Zemunik et al. 2020), in cases where these mechanisms are not sufficient, the plants are inevitably stressed by excess Mn and therefore weakened towards environmental threats, such as droughts or pathogens. This handicap has been attributed to the metabolic costs of compartmentalising Mn in the vacuoles (Fernando and Lynch 2015; Wills et al. 2023). The threshold of Mn FECs for the sustainable growth of sugar maple (*Acer saccharum* Marshall) in N America is approximately 2500 mg kg^−1^ (Kogelmann and Sharpe 2006), whereas such a limit has not yet been determined for birch. The highest Mn FEC concentrations that have been found in birch leaves in Litvínov are approximately 8000 mg kg^−1^ (Wildová et al. 2021), and in samples from two trees that were sampled at the same locality (MKB subset) in September 2022, we found concentrations between 4000 and 4500 mg Mn kg^−1^. The leaves of these trees were relatively small and deformed, and the tree crowns were thin, demonstrating their limited health. Therefore, we consider that birch Mn FECs between 5000 and 8000 mg kg^−1^ are generally excessive. With respect to the other study areas, only one specimen from the Beskid Mountains and two from the Harz Mountains fell within this interval.

## Conclusions

The Mn FECs in birch trees result from an interplay of geological, geochemical, pedogenic, topographic, and anthropogenic controlling factors. Mn FECs are elevated in cases of low macronutrient uptake (Mg, occasionally also K), for trees growing on soils with felsic or silicic bedrock, and in soils impacted by acid rain. Thus, high-Mn FECs in birch leaves can be used as diagnostic tools to map the consequences of soil acidification. Furthermore, this study documents the downslope transport of Mn in persistent acid soil. Finally, high Mn FECs are systematically associated with elevated concentrations of Zn FECs, which is interpreted as (i) the joint impact of soil acidification on both elements and (ii) Zn input associated with the metallurgy of iron, steel, and non-ferrous metals, all jointly impacting and damaging soils. Therefore, the consequences of anthropogenic soil acidification can be examined by mapping birch FECs as they reflect the actual soil geochemistry in the root zones of the studied trees. The highest Mn FECs in birch leaves found in this study were near the expected limit for healthy tree growth, estimated to be > 5000 mg kg^−1^.

### Supplementary Information

Below is the link to the electronic supplementary material.

Supplementary file1 (PDF 2381 KB) Fig. S1. Contamination sources around the Thüringer Wald and Harz mountains and birch leaves sampling sites.

Supplementary file2 (XLSX 14 KB) Table S1. FECs from seasonal sampling in the Beskid Mountains, subset VH.

Supplementary file3 (XLSX 14 KB) Table S2. FECs from seasonal sampling in the Ore Mountains, subset MKB.

Supplementary file4 (XLSX 36 KB) Table S3. FECs from Central European sampling subset GB.

## Data Availability

Datasets of Mn concentrations (table_S1and table_S2) and datasets of Mn, Mg, P, K, Ca, and Zn concentration (table_S3) in leaves from Central European samples are uploaded as supplementary files. Norwegian datasets can be found in references to the individual transects. Corresponding authors could provide datasets with other examined elements on request.
