# Supplementary material for: Factors controlling Mn and Zn contents in leaves of silver and downy birch in acidified soils of Central Europe and Norway
Source: Environ Sci Pollut Res Int. 2024 Jan 9;31(6):9642–60. doi: 10.1007/s11356-024-31837-w (PMC10824807; doi:10.1007/s11356-024-31837-w)

Environmental Science and Pollution Research

Factors controlling Mn and Zn contents in leaves of silver and downy birch in acidified soils of central Europe and Norway

Gabriela Bílková[1,2,*], Michaela Königová[1], Věra Hýlová[3], Jitka Elznicová[1], Hans von Suchodoletz[4], Belinda Flem[5], Tomáš Matys Grygar[2,*]

[1] Faculty of Environment, J. E. Purkyně Universtity in Ústí nad Labem, Pasteurova 15, 400 96 Ústí nad Labem, Czech Republic

[2] Institute of Inorganic Chemistry of Czech Academy of Sciences, 250 68 Řež, Czech Republic

[3] Náměstí Generála Svobody 985/23, 700 30, Ostrava, Czech Republi

[4] Geoinformatics and Remote Sensing Group, Institute of Geography, Leipzig University Johannisallee 19a, D - 04103 Leipzig, Germany

[5] Geological Survey of Norway, POB 6315 Torgarden, N-7491 Trondheim, Norway

* corresponding authors, Gabriela.Bilkova@ujep.cz, grygar@iic.cas.cz

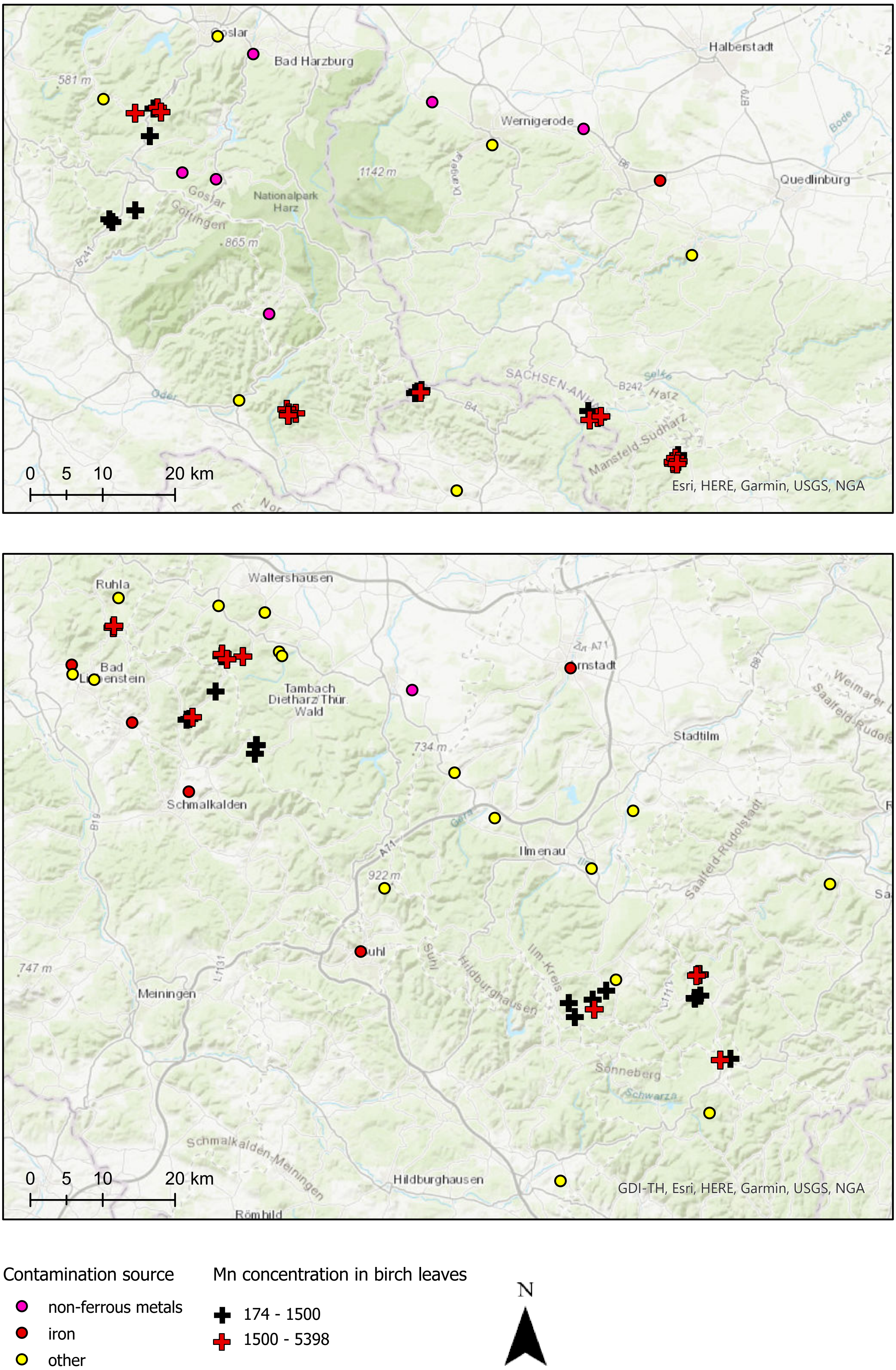

Supplement: Supplementary file 1 — Supplementary file1 (PDF 2381 KB) Fig. S1. Contamination sources around the Thüringer Wald and Harz mountains and birch leaves sampling sites. [file 11356_2024_31837_MOESM1_ESM.pdf]
